# Telling Ecopoetic Stories: Wax Worms, Care, and the Cultivation of Other Sensibilities

**DOI:** 10.1007/s10912-024-09878-6

**Published:** 2024-08-15

**Authors:** Martin Grünfeld

**Affiliations:** Department of Science Education, University of Copenhagen, Copenhagen, Denmark

**Keywords:** Artistic research, Care, Ecopoetics, Materiality, Posthumanities, Wax worms

## Abstract

Recently, a beekeeper discovered the metabolic wizardry of wax worms, their ability to decompose polyethylene. While this organism has usually been perceived as a model organism in science or a pest to beekeepers, it acquired a new mode of being as potentially probiotic, inviting us to dream of a future without plastic waste. In this paper, I explore how wax worms are entangled with material practices of care and narratives that give meaning to these practices. These stories, however, are marked by manipulation, exploitation, and extermination, and call for a questioning of our modes of caring. Consequently, I offer a counter-narrative that questions our anthropocentric practices of caring and the stories we attach to them. Borrowing Puig de la Bellacasa’s notion of *ecopoetics*, I tell another story based on my participation in the making of an art installation hosting wax worms. The installation creates an opening of a world of curiosity and cultivates a sensibility for wax worms expanding their modes of being and our capabilities of appreciation. In the end, I argue that by mattering and storying differently, we have the opportunity to challenge anthropocentric interests and make a different world of caring and co-existence possible.

## Introduction

When we behold a wide, turf-covered expanse, we should remember that its smoothness, on which so much of its beauty depends, is mainly due to all the inequalities having been slowly levelled by worms. It is a marvellous reflection that the whole of the superficial mould over any such expanse has passed, and will again pass, every few years through the bodies of worms. The plough is one of the most ancient and most valuable of man’s inventions; but long before he existed the land was in fact regularly ploughed, and still continues to be thus ploughed by earthworms. It may be doubted whether there are many other animals which have played so important a part in the history of the world, as have these lowly organized creatures. (Darwin 1945, 148)

In his book on English earthworms, Charles Darwin pays tribute to their important part in the history of the world. As Darwin highlights, earthworms “garden” the soil, making vegetable mold for even the choiciest plants to grow in. In this process, these ancient caregivers transform the earth, making it hospitable to humans. Or as Bennett (2010, 96) notes, the small agencies of earthworms in Darwin’s narrative have big, accumulated effects on world history inaugurating and sustaining human culture. No wonder, then, that Darwin ends his book with such a grandiose tribute to earthworms. Recent soil science also attributes great powers to earthworms as they turn dirt into fertile soil as ecosystem engineers transforming the composition and activity of microorganisms responsible for decomposing natural substrates (Lavelle 2000; Stewart 2004). Within the humanities and social sciences, earthworms (and other ecosystem engineers) have also received increased attention because of their capabilities to transform the soil beneath our feet and contribute to the health of our planet. For example, Latour (1999, 74–76) follows soil pedologists in the Brazilian Amazon Forest, exploring the role of earthworms transforming the boundary between savannah and forest; Abrahamsson and Bertoni (2014) discuss the messy yet productive practices of composting with earthworms through their own attempts to live together with these more-than-human organisms, and Puig de la Bellacasa (2017, 214) argues for a more-than-human perspective on soil that takes the livelihood of earthworm communities seriously up against the accelerated yield that fertilizers provide. Although this paper draws inspiration from these studies, it also changes the perspective from the underground care carried out by earthworms to wax worms—the larvae of the greater wax moth (*Galleria mellonella*)—and their multifaceted entanglement with human caring practices.

By now you might very well wonder: why tell stories about wax worms in a special issue on narratives and materialities of care? Why are they so special that we need to devote our attention to them? While we have long known the small but important agencies of earthworms, wax worms have existed alongside us mainly being exploited as model organisms and being exterminated as pests threatening the fragile ecosystem of bees (not to mention being used as a delicious fatty-rich snack for pet lizards). Yet recently, wax worms have found their way to the headlines of scientific magazines and newspapers, e.g., “This plastic-foam-eating ‘superworm’ could help solve the garbage crisis” (Verma 2022). What interests me in this paper is how this serendipitously discovered potential for bioremediation changes the narratives of wax worms, while the correlative material practices of care are reshaped in-between natural habitat and laboratory settings. By retelling stories about wax worms as a *model organism*, *pest*, and *probiotic hero*, I wish to unfold how their different modes of being are embedded within different but related narratives. To do so, I focus on how these modes of being are framed within popular and scientific discourse, especially focusing on the stories wax worms participate in. I use *story* here to designate the narrative content within which they act, passively or actively (Genette 1990, 27). However, rather than importing an entire narratological framework to analyze texts on wax worms, I focus specifically on wax worms as *actants*, i.e., the positions and functions of wax worms as a class of actors and how they are acting and acted upon within caring practices (Bal 1997, 195–207). Here it is also useful to think about how *actants* have been defined broadly by Latour (1996) as anything that is granted to be a source of action without intention, no matter if we are talking about humans, animals, or doors. In this reframing, actants become ontological hybrids that make worlds (Latour 1996). In this paper, wax worms appear within material relations of care described in narratives that shape the way we perceive them and the affordances they grant us.

A key focus in this paper revolves around the ambiguous and troubled notion of *care*. As Tronto (2015) notes, care-taking practices are often unrecognized or invisible. When we pay attention to practices of care, however, we easily find them in multiple situations from private homes to institutionalized care units, from airplanes to museums, and from gardening to laboratory research. Yet these multiple modes of caring do not necessarily fit neatly within one definition of care but rather as Murphy (2015) argues fall within (at least) four different modes of caring: from emotional attachment or nurturing to being attentive or worried. But the notion of *care* is not just troubled by the multiple modes of caring; rather, as Law (2010) argues, in practice, caring involves multiple objects that change positions over time in situated actions. Based on Law’s insights, I focus on how the modes of being of wax worms diverge in different localities that shape the material practices of care as well as the narratives that give meaning to these practices. In doing so, I draw on the recent discussions of how care can be deeply ambiguous even involving violent forms of engagement and multiple feelings not just positive feelings (Murphy 2015; Puig de la Bellacasa, 2017, 1). As Mol et al. (2010) argue, we must pay attention to care in specific practices and sites. Yet, as I focus on stories that unfold material caring relationships with wax worms across multiple sites, what is particularly interesting to explore in this paper is how the situated caring relations are correlative with their multiple modes of being (as model organism, as pest, and as probiotic), yet retain wax worms as entities that can reasonably be manipulated, exploited, and killed. Even after being discovered as potentially probiotic heroes in the sense that they can be appropriated to cure or heal planetary issues of pollution (Lorimer 2020, 2), wax worms remain killable entities.

Perhaps this discrepancy calls for other *ecopoetic* stories. In her recent work, Puig de la Bellacasa (2021, 198–99) develops an *ecopoetics* that blends ecological thinking, defined as the interdependent interactions between multiple forms of life, with poetics to call upon the powers of language, metaphors, and stories to make consequential ecological involvements. To do that, it “matters what stories we tell,” she continues. However, as Morton (2010, 7–8) notes, ecological thought is not just about ecology but itself a thinking that is ecological and, thus, deeply relational, intertwined, and messy. The *worm stories* (a term borrowed from Bennett 2010, 94) I explore in this paper are intended to open a sprawling mess that relates by way of a recurrent actant—wax worms—to unfold a multiple ontology of complicated material relations of care. Paying attention to these stories allows us to understand how they shape and are shaped in material caring relations (Buse et al. 2018). Yet they also call us to question our perception of these organisms and perhaps develop other ways of caring that decenter the human.

To cultivate other ways of caring, I propose a counter-narrative that tells another story based on my transdisciplinary collaboration with sound artist Eduardo Abrantes and conservator Amalie Schjøtt-Wieth in the making of an art installation hosting wax worms. Similar to their surfacing within academic research across disciplines, worms are also increasingly thematized in art. Within the contemporary art world, worms are heralded as heroes and transformers of the earth, for example, in Alice Channer’s exhibition *Worms* (2021) and Tamara Henderson’s solo exhibition *Green in Grooves* (2023). Yet what characterizes these exhibitions is how the transformative powers and deeply embedded nature of worms are materialized in the displayed artworks and influence artistic practice, while actual living worms are not part of the exhibitions. An interesting exception is the immersive installation *Mass* (2018) by the Recoil Performance Group, in which a performer and thousands of mealworms live together inside a dome made of plastic. Over time, this transgressive worm performance alters the space as the performer moves within it and the worms digest the dome (Recoil Performance Group). The slow dissolution of the installation highlights not just the capabilities of mealworms as composters but also thematizes the vulnerable relationship between worms, plastics, and humans in a polluted world. Similarly, the alternate ecopoetic story that I will unfold in this paper describes our attempt to craft a material opening of a world of curiosity to cultivate a sensibility for wax worms expanding their modes of being and our capabilities of appreciation and co-existence.

My aim in this paper is thus twofold. First, I retell three stories of the caring relations between wax worms, humans, and the environment (in the “Telling worm stories: Wax worms and the multiple materialities of care” section). Second, I contrast these stories with an eco-poetic counter-narrative that through artistic practice challenges our habitual caring relationships and narratives in order to cultivate a material sensibility for the more-than-human wax worms as co-inhabitants on this planet (in the “Storying and mattering differently: Of care and co-existence” section). In the end (the “Conclusion: Towards an expanded notion of care” section), I argue that *mattering* and *storying* differently is an opportunity to dissolve anthropocentric interests and dream of not only a world without plastic waste but a different world of caring and co-existence.

## Telling worm stories: Wax worms and the multiple materialities of care

In this section, I tell three stories of wax worms—stories that aim to encapsulate some of our usual ways of engagement with wax worms in Western culture and science. These narratives are unfolded, however, not only to exemplify how we engage with wax worms but also to capture their modes of existence as actants within networks of objects, practices, interests, etc., in other words, relationships that tell us about the material practices of care they are embedded in. Telling such stories has a double purpose. First, telling stories of wax worms gives attention to an often (and until recently) neglected organism entangled in human culture. Second, telling such stories is also *telling* of our multi-faceted relationships with this organism and our caring engagements. I have chosen three common narratives of wax worms that present different, even conflicting modes of existence. First, I tell the story of wax worms as a model organism used in antimicrobial laboratory studies of pathogens. This is a story of usefulness and exploitation that I contrast with a second story of wax worms as an environmental pest and ecological antagonist threatening bee populations, even entire ecosystems. Meanwhile, in the third story, I focus on how wax worms recently have changed status from being either a convenient model organism or a threatening pest to becoming a potential probiotic hero inviting us to dream of a future rid of plastic waste. After these three stories, I compare how they frame the modes of being of wax worms and their roles within practices of care.

### Being a model organism: The convenience and care of wax worms in the lab

Galleria mellonella (greater wax moth or honeycomb moth) has been introduced as an alternative model to study microbial infections. G. mellonella larvae can be easily and inexpensively obtained in large numbers and are simple to use as they don’t require special lab equipment. There are no ethical constraints and their short life cycle makes them ideal for large-scale studies. Although insects lack an adaptive immune response, their innate immune response shows remarkable similarities with the immune response in vertebrates. (Tsai et al. 2016)

When you read introductions to studies utilizing wax worms, you very often encounter the narrative above. It tells a story of the recent popularity of wax worms as a model organism especially in the study of microbial infections and antimicrobial compound testing. As the excerpt outlines, wax worms are convenient for both practical and epistemic reasons: they are cheap, simple to use, and have a short life cycle, which makes them ideal for large-scale studies, and they have an innate immune response with components homologous to the human innate immune system making them suitable for studying microbial pathogens (Pereira and Rossi 2020). As Leonelli and Ankeny (2013) note, model organisms must have experimental characteristics that work alongside their genetic powers, such as low-cost breeding, maintenance, and transport, as well as short life cycles, high fertility, and mutation rates. These practical attributes are crucial for the success of model organisms. Furthermore, wax worms are also convenient ethically as they can be used almost without any ethical formalities because invertebrates are excluded from the *Animal act of 1985* (Andrea et al. 2019; Wojda et al. 2020).

Yet this story of wax worms also comes with a challenge. Like other model organisms, the success of the model depends on standardization, and, currently, there are discrepancies across diverse laboratories using wax worms (Andrea et al. 2019). This is a point where epistemic aims and caring engagements intertwine. Indeed, the well-being of model organisms is crucial for producing high-quality science and how laboratory animals are cared for within laboratory settings matters for biomedical knowledge production (Friese and Latimer 2019). Lab technicians care for the well-being of wax worms through a material set of practices that include feeding them, moving them between containers, cleaning containers, and monitoring their melanization responses to their living conditions and experimental inoculations. In laboratory settings, wax worms go through their lifecycle from egg to moth in three different containers of either plastic, glass, or metal, one for each of their stages at temperatures between 25 and 37 °C (Pereira and Rossi 2020). In these controlled settings, temperature, light, and humidity all play a role in the breeding and housing of wax worms as they—together with food—impact their size and immune system development. These attentive practices of care, however, only focus on the well-being of wax worms for higher purposes of developing drugs and treatments for human patients that the organism can be sacrificed in the name of. Consequently, as model organisms, wax worms are attentively cared for to be useful in caring for human health.

### Being a pest: Wax worms as ecological threats and killable entities

The greater wax moth, Galleria mellonella Linnaeus, is a ubiquitous pest of the honeybee, Apis mellifera Linnaeus, and Apis cerana Fabricius. The greater wax moth larvae burrow into the edge of unsealed cells with pollen, bee brood, and honey through to the midrib of honeybee comb. Burrowing larvae leave behind masses of webs which causes galleriasis and later absconding of colonies. The damage caused by G. mellonella larvae is severe in tropical and sub-tropical regions, and is believed to be one of the contributing factors to the decline in both feral and wild honeybee populations. (Kwadha et al. 2017)

Outside laboratory settings, wax worms take on a very different role as a pest known to infest beehives around the globe. Bees, both wild and managed, are widely acknowledged as crucial agents in the maintenance of healthy ecologies. They are providers of cultural and ecosystem services by, e.g., pollinating crops impacting both economies and health and sustaining populations of birds impacting biodiversity and ecosystem stability (Klein et al. 2018). Yet everywhere beekeeping is practiced, wax worms infest bee colonies from the inside: Moths enter hives during the night and lay their eggs in crevices (Parepely et al. 2023). As eggs hatch and tiny but hungry larvae emerge, the bee colony is under threat of collapse as the larvae eat pollen, honey, and beeswax, while creating silk-lined tunnels through the hive and holes through which honey may leak out (Kwadha et al. 2017). In such situations, beekeepers find themselves managing infestations essentially monitoring, cleaning, and maintaining beehives and attempting to exterminate wax worms.

Here, care and killing go together when an unwanted antagonist such as wax worms threatens ecosystem stability and planetary health as well as human economic and cultural interests. Care and killing may sound like opposites but as Law (2010) shows in his study of the objects of care involved in the culling of animals in Devon during UK’s foot and mouth epidemic, care and killing can also go together in uncertain choreographies of care. However, contrary to Law’s description, the extermination of wax worms in beehives leads to no ambivalences. Due to lacking ethical regulations and their potentially devastating impact on ecosystem health, wax worms are genuinely killable entities, and their extermination from beehives leaves no traces of doubt: the means (killing invertebrates without rights) satisfy the ends (increased ecological variation, pollination, and planetary health). Doubt can be found mainly concerning the question of methods for extermination. While some methods resemble laboratory practices such as cleaning, moving, and monitoring temperature, they are utilized in reverse with the aim not to sustain but to exterminate entire populations of wax worms (“BeeAware”). Chemical fumigants are also being used to manage the threat of pest infestations in beehives. Yet chemical treatments utilizing, for example, calcium cyanide, methyl bromide, and carbon dioxide pose serious health risks for the handler and the consumer as they may lead to residues building up in hives and honey as well as being poisonous to honeybees these extermination methods are supposed to protect (Kwadha et al. 2017). This raises serious dilemmas not in terms of the objects under extermination but rather the effects of the various interventions: you can apply small-scale cultural practices of care including removing and cleaning with relatively limited effects or you risk killing populations of not just wax worms but also non-target organisms including bees. Consequently, scientists currently seek to develop sustainable biological control agents such as bacteria and fungi that target wax worms directly without toxic side effects, but so far, it looks like the hungry wax worm and ecological antagonist is likely to extend their stay in beehives despite efforts to care for the environment through management, control, and extermination.

### Becoming probiotic: A *hero*’s tale from wax worms to enzymes

The tiny waxworm went from zero to hero in 2017 when researchers discovered the caterpillar could potentially help solve one of the world’s most pressing environmental problems: plastic waste. (Hunt 2020)

From 2017 and onwards, wax worms became a frequent subject in newspapers and popular science magazines. An amateur beekeeper—Federica Bertocchini—serendipitously discovered something that radically changed our perception of it. She discovered their ability to decompose plastic (polyethene or PE) when she was tending to her beehives and noticed that they were infested with wax worms. Following the cultural beekeeping practices described above, Bertocchini essentially cleaned the honeycomb and captured the wax worms in a plastic bag only to realize their metabolic wizardry a few hours later, when they had eaten their way out and were everywhere in her apartment. As Bertocchini described the situation: “After a while, I noticed lots of holes and we found it wasn’t only chewing, it was [chemical breakdown], so that was the beginning of the story” (Bertocchini). A very different story not only for the discoverer, who is also a scientist now leading the research and development of biotechnological solutions to plastic waste, but also a story unraveling a completely different mode of being for wax worms entering other caring relations and bringing them into public attention.

Could wax worms then become the probiotic hero we have been waiting for as plastic waste piles up? *Probiotic* as a concept here is borrowed from Lorimer (2020, 2) to designate “human interventions to use life to manage life, working with biological and geomorphic processes to deliver forms of human, environmental, and even planetary health.” As Lorimer notes, the probiotic is about much more than a preference for live yoghurt; it is an effort to change the composition of biophysical systems enlisting selected organisms and eventually engineering them to assist us in bioremediation and waste management. Indeed, the metabolic capabilities to deteriorate plastic (PE) could provide the small agency of wax worms with potentially huge accumulated effects helping us to manage and decrease plastic pollution. Plastic poses serious ecological issues as millions of tons are dumped every year, entering ecosystems from the summit of Mount Everest to our bloodstream, where microplastics are becoming part of our flesh (Alaimo 2014; Carrington 2022). While wax worms have developed the capabilities to degrade plastics due to the similarities between the composition of beeswax and polyethylene, their metabolic wizardry led to a scientific race to figure out how to develop a natural solution to degrade plastic waste (Bombelli et al. 2017; Goñi 2017).

This also brings our story back into laboratories where wax worms were dissected and killed, even mashed, homogenized, and smeared onto plastics in efforts to find the key to their abilities (Bombelli et al. 2017). In late 2022, researchers made the groundbreaking discovery that a key factor in their polyethylene degradation lies in enzymes in their saliva (Sanluis-Verdes et al. 2022). By collecting saliva from the mouths of live wax worms and exposing PE film to it for a few hours at room temperature, the researchers found effects of oxidation equivalent to months or even years of weathering. This led to another reappearance on front covers with headlines such as: “Wax Worm Saliva is the Unlikely Hero of Fighting Plastic Waste” (Germain 2022). In the saliva, particularly two enzymes turned out to play a key role in the biodeterioration of PE. As mythological goddesses of the earth, the two enzymes aptly named *Ceres* and *Demetra* appeared on stage to tend to an earth overpopulated by polymers (“Plasticentropy.Net”). Currently, researchers are working to scale up the capabilities of these enzymes to deal with the massive pollution of plastic globally. Yet it has also been questioned whether this solution is viable given that PE is a high-quality resin that can be upcycled (Arnold 2017). Moreover, we can also question whether such solutions will only lead to legitimizing throwing away even more plastic waste, rather than taking care of the environment. While the humble wax worm returns to the darkness of artificial larvae colonies maintained at 28°Celsius in lab settings not unlike its model organism siblings, *Ceres* and *Demetra* perhaps prepare the way for biological solutions to the massive plastic pollution the world is currently facing.

### Narratives of care: What the worm stories tell us

In the stories I have retold here, the modes of being of wax worms vary from model organism to pest and potential probiotic being. As a model organism, wax worms are essentially acting as a helper or remedy in studies of anti-microbial drug compounds. Meanwhile, in the second story, wax worms embody an active and materially destructive agency as an ecological antagonist threatening bee populations. The third story counters this agency as an antagonist by highlighting the probiotic potential of wax worms to deteriorate polyethylene. Despite the difficulties involved in specifying what a hero is, the third story could be a hero’s tale, because of the functional characteristics that wax worms only possess and their potential to overcome the serious plastic pollution threatening planetary futures (Bal 1997, 132). Yet the third story is also a story of how the hero always becomes smaller in science. As the story progresses, their mode of being begins to change from a potential savior of planetary health to a more modest role (not unlike the mode of being a model organism) as a material basis for even smaller heroic agents—the enzymes Ceres and Demetra.

The different modes of being explored here also entail correlative differences in the practices of care. The three stories take place in different settings that configure the caring relations differently (Buse et al. 2018). As model organisms, wax worms are cared for in controlled lab settings and linked to material practices of care involving containers, incubators, cleaning, and maintenance, but always with epistemic purposes in mind. This is a deeply anthropocentric care practice that provides for wax worms while deliberately infecting them as pathogenic hosts to exploit their innate immune responses. Meanwhile, in natural settings, wax worms threaten bee colonies, and beekeepers aim their caring practices *against* wax worms focusing on maintenance and cleaning as well as chemical extermination. Similar to Law’s study of the choreography of care, we could say that killing becomes an act of care for the sake of the bigger picture (Law 2010, 64), in this case, to protect not just economic interests but also environmental health. Similar to the second story, the third story is also about environmental health albeit in reverse as wax worms take on a new role as a potential probiotic hero. The story blends cultural practices of beekeeping with laboratory techniques for hosting and experimenting with wax worms. When the natural and laboratory settings mesh, something new emerges—the potential of another organism saving us from our pollutive ways of living. Yet while the two latter stories put emphasis on planetary health, their caring practices remain caught within a web of anthropocentric interests spun so explicitly in the first story.

## Storying and mattering differently: Of care and co-existence

The worm stories we have encountered so far showcase different modes of being within correlative material practices of care marked by exploitation, extermination, and exploration. Even within broader explorative modes of caring for the environment, however, the human remains centered as the main actant and beneficiary, while wax worms are constructed as killable entities worth sacrificing for higher purposes. But what if we begin to question such self-given species differences? Taking this discussion does not entail that we cannot use wax worms in laboratory research (or must stop exterminating wax worms from beehives), but rather as Haraway aptly notes that such practices should never leave us in moral comfort (2008, 75). Following Haraway, we have an ethical obligation to keep the inequality between human and non-human from becoming self-given (2008, 77 & 84). Beyond taxonomic hierarchies and sacrificial us/them logics, can we perhaps cultivate a radical ability to feel and respond to other beings in an irreducible world of embodied and partial differences (Haraway 2008, 75)?

In this section, I retell another worm story that functions as a counter-narrative—an ecopoetic story that tries to make consequential ecological involvements. But while it matters which stories we tell, to truly engage we must embrace the difficulties of caring *for* other organisms and blend *storying* and *mattering*. In contrast to the other stories, I am directly implicated in this story together with my collaborators, conservator Amalie Schjøtt-Wieth and artist Eduardo Abrantes. The story is about the making of an art installation hosting wax worms in an experimental exhibit called *The Living Room* housed at the *Medical Museion *in Copenhagen. In *The Living Room*, we attempted to develop a posthuman practice of care that engaged with, instead of suppressing, living organisms embracing even cultivating transformations at the museum (Grünfeld 2022; Grünfeld et al., 2023). In this context, we became particularly interested in wax worms because of their metabolic wizardry, and they became the protagonist in our art installation entitled *Worm dome*. We wanted to craft a material world that could do justice to their amazing capabilities and probiotic potentials—a justice ingrained in the materiality of being that unmakes absences and supports alternate assemblies (Papadopolos, 2014). Instead of being sacrificed in anonymity, we wanted to create an alternate world of curiosity and appreciation. It is this ecopoetic story of an alternate assembly I will now tell.

### Becoming art: Wax worms as decomposers and composers

We invite you to lie down and crawl with them. To imagine your every movement as involving your whole body, flexing, and slowly pulsating, squirming forward. You come close to and mesh with foreign bodies and with foreign objects – the warmth of flesh wrapped in clothes, the warm hardness of wood, the flexibility of plastic, the cool hardness of metal and glass, the stickiness of your fingers and mouth, exploring, sensing – learning the environment around you. (Abrantes 2021a)

November 4, 2021. We are roughly 70 people lying on the floor wiggling like worms. Wax worms take a central position on a table in the middle of the room inside a desiccator turned into an art installation. Desiccators are glass containers usually used to extract humidity from objects. Inside the desiccator, we have arranged plastic materials collected from a local lab mixed with other disposable objects and honeycomb for the worms to eat as well as analog sensors registering humidity variations, CO2 levels, and vibrations extracting this data from the worms and habitat and transforming it into sound. Sonification, in this case, grants us a chimeric sense of the inaudible metabolic processes and movements of the wax worms inhabiting the installation (Abrantes 2021b). The sounds are transmitted live to the mobile phones of the participants and provide the soundscape we are moving to. Despite our large bodies and radically different sensorial makeup, we nonetheless attempt to assume a worms-eye view or rather an embodied experience of a mode of being that is much more directly entangled in the world than our own. In comparison to the transgressive durational performance *Mass*, in which the performer and potentially one visitor at a time entangle inside a large plastic dome encountering mealworms directly (Recoil Performance Group), our engagement is less bodily entangled but perhaps more communal. What we touch while lying on the floor is not the worms nor their habitat, but Eduardo complements our embodied yet distanced engagement with a performance lecture to unfold connections between wax worms, objects, and ourselves. The imaginative enactments and embodied listening stitch together bodies beyond individualized organisms—both the visitors wiggling in communion and the more-than-human worms in an intermediate zone (Grünfeld 2024). Through this art for noticing, we try to cultivate a sensibility for invertebrates that may appear to almost overturn the power to the worms bringing their Otherness to the center of attention and appreciation in a museum space usually devoted to dead things.

Yet while our performative experiment may sound like an optimistic attempt at cultivating sensibility and co-existence across alleged ontological differences, our experiment also carries along a darker ecology of horror and uncertainty (Morton 2010, 17). Although we were trying to create a tiny circulatory ecosystem with thriving larvae and swirling moths reproducing the next population of plastic munching larvae, in reality, a few months after their arrival, we had a decomposing ecosystem at our hands. At the bottom of the desiccator, we placed a mesh of food that gradually began decomposing. Paradoxically, our attempt to provide for the worms turned into its opposite as a toxic environment, and the worms gradually turned blackish—a clear sign that they were dying. Then, on a random Friday morning in April 2022, Eduardo and I first noticed grayish-brownish debris on the wooden plinth on which the desiccator was resting. When we zoomed in on the debris with a microscope, the debris was teeming with living tiny sugar mites (presumably *Lepidoglyphus destructor*) breaching the crucial boundary between the ecosystem and the museum. As we enveloped the desiccator in two plastic bags to secure the museum site, our attempts at a posthuman practice of care became unsettled and troubled by the material paradoxes of our own making.

### Significant, yet significantly other: Cultivating sensibilities and the question of anthropomorphism

What does this counter-story tell us about wax worms and how does it compare to the other worm stories? If we begin by contemplating how the mode of being of the wax worms is reorganized within the installation we call *Worm dome*, we discover an intermeshed network of multiple modes of being already encountered in the other stories. Like a model organism, the worms in the *Worm dome* are contained and provide a material basis albeit not for research but for artistic experimentation. Yet we did not have the infrastructure to breed populations of wax worms similar to the lab setting, but instead attempted to create a livable habitat bringing elements relative to its other modes of being (naturally sourced honeycomb and polyethene). This is one opportunity that artistic practice offers: the opportunity to explore their multiple modes of being, rather than reducing the existence of an organism for the sake of epistemic clarity or effective pest management. Moreover, although the worms may appear to be captive in the installation, they are also materially engaged in transforming the installation as a composting-composing protagonist living (and dying) as art. Although the story has a tragic ending, when the composter-composers are themselves decomposing and other organisms emerge, the wax worms were our significantly unfree partners (Haraway 2008, 72) that we tried to relate to. For Haraway, caring entails becoming subject to an unsettling obligation of curiosity, and we attempted to unfold and maintain this moment of curiosity in the engagement with the wax worms and with visitors. An unsettling obligation that is both attentive and troubling and shows how the nicely laid out modes of caring that Murphy (2015) develops can also become entangled (Grünfeld 2024). Yet, as Haraway notes (2008, 90), caring for more-than-human animals is easier when we are relating to vertebrates with sizable brains and human similarities. Despite their innate immune response homologies with humans, wax worms (and other invertebrates) call for a nonanthropomorphic sensibility accountable for the irreducible differences. Yet how do we care for such significant Others and is our story bringing us any way near that or are we just stuck in anthropomorphic ontological play?

If we look more closely at the practices of care involved in our artistic experiment, they differ significantly from the other worm stories retold in this paper. As Cameron points out, in a more-than-human world, museums are ideally placed to promote posthuman theories and practices of life (Braidotti and Hlavajova 2018, 349). Going beyond the usual anthropocentric stories, perhaps surprisingly, the almost always too-human museum can establish a contact zone facilitating weird encounters with the more-than-human (Grünfeld 2021). In this setting, our installation of wax worms invites ourselves and our visitors to engage differently with a significantly other organism. This setting becomes an in-between space that overturns (a bit of) control and power to the wax worms as decomposers in the process of becoming composers of a soundscape that calls for a non-mimetic sensibility embodied in all too human forms during the performance lecture. This inevitable anthropomorphism unfolds as we attempt to mimic their movements and describe their forms through anthropomorphic forms of engagement. Rather than going all the way to cultivating a nonanthropomorphic sensibility that Haraway calls for, this is the curse (or blessing) of being human and why we still also need stories. Indeed, as Bogost (2012, 64) argues, we cannot do without anthropomorphism, but this is not a problem, but rather a creative resource. A creative resource that is not limited to the human but as Bennett (2010, 99) notes is also a sensibility that uncovers the world partially and situationally (Bennett 2010, 99). As we care for the wax worms through an art of troubled noticing, our sensory experience of their materiality impacts us as careers (Buse et al. 2018). We are co-formed by them in our desire to care, yet our relationships remain troubled and ambivalent.

Our artistic experiment is at once an ontological play crafting alternate assemblies and a serious cultivation of a sensibility for the significant Other. Borrowing from Bogost, we might call this a speculative alien phenomenology drawing partly on spoken word and partly on sonified sounds as material planes for our encounter with these worms at the limits of our experiences. A paradoxical engagement at once attentive and distanced relying on unfaithful modes of listening and mimicking their movements. An attentive engagement that slows down to notice the tiny agency of other organisms and troubles our polluting ways of living. In the Anthropocene, we are the polluters, plagues, and pests, and we must overcome our destructive ways of life for planetary health. Perhaps then we might begin to question what stories we tell and our self-given material practices of care.

## Conclusion: Towards an expanded notion of care

This paper has been teeming with worms taking multiple forms and functions from docile model organisms in labs to unruly environmental pests in the wild. My aim has been to retell such stories to explore how wax worms embody different modes of being within Western culture. The stories, however, are not just discursive but also tell of the divergent material relations of care involving human-wax worm relations. As I unfold their multiple modes of being, we see not just how they exist as a model organism, a pest, and a probiotic being, but also how their modalities are shaped by mundane and technical equipment used in the material practices of care and the locations shaping these practices. In the lab setting, wax worms exist as two dissimilar objects of care: through the mundane practices of housing, feeding, and cleaning, wax worms are reared to thrive within artificial settings until the moment when they are exploited and infected as pathogenic hosts and become part of a larger infrastructure of care that aims to develop future anti-microbial drugs. As a model organism, wax worms essentially exist as a remedy for research, in contrast, to their unruly effects in the wild. If there are tensions involved in the duality of caring for and killing wax worms as a model organism, this is not the case in the wild where they threaten bee populations as an ecological antagonist unwelcome in its own habitat. Consequently, caring and killing travel together, and beekeepers care *against* wax worms utilizing cultural practices of care involving maintenance and cleaning as well as chemical extermination efforts. Yet it is precisely, and perhaps paradoxically, through such beekeeping practices of care that a completely new mode of being for wax worms emerges as potential probiotic heroes. This is where wax worms now enter world history in a similar fashion as Darwin’s earthworms, as a potential hero with metabolic capabilities to transform the earth. However, an earth, very different from Darwin’s time, ravaged by human habitation, overproduction, and pollution. Though the modes of being of wax worms diverge and the correlative practices of care range from mundane rearing or extermination to the benefit of humanity and planetary health, wax worms remain caught within anthropocentric interests. In other words, care in the context of wax worms is always directed towards anywhere else than the worms.

What if we unmake their absences and direct our caring engagements towards wax worms? In this paper, I have not just explored narratives of wax worms and correlative material practices of care, but also offered an ecopoetic counter-narrative that, following Puig de la Bellacasa, disrupts the individualized human-centered notions of agency and responsibility. Based on our development of the art installation *Worm dome* and a subsequent performance treating wax worms as protagonists, I offer a counter-narrative that shows us how we might cultivate our sensibilities for other organisms and care differently through an art of noticing: an art of noticing that listens to the inaudible metabolic processes of decomposing plastic waste and enacts alien bodies in too human forms. This attentive mode of care becomes almost reciprocal as we are providing for wax worms, while entering a circulatory web of caring relations that impacts our own bodies, senses, and thoughts, however temporary such experiences may be. By storying and mattering differently, we craft ontological openings into the significantly other world of wax worms as they become art calling upon us another sensibility. However, as they encounter the plastic by-products and live their lives as art, the wax worms in the installation do not reproduce as hoped for, but rather leave us with a tiny decomposing ecosystem turning into an ironic monument of our own failures to provide and care for these worms, yet also affirms life’s ability to persist as new organisms emerge. This duality carves out a tension ingrained in our practice between celebrating the indeterminacy of life and our responsibilities as actors within lively relationalities. Our caring relations impact both wax worms and ourselves opening an ontological space for co-existence with these organisms without rights. Yet our caring relations are also paradoxically attentive yet distanced and troubled engagements that do not solve environmental issues but decelerate to attune ourselves to the tiny agency of wax worms and the vulnerability of matter as it circulates and exchanges. While the counter-narrative offered here does not solve the environmental issues that wax worms treated as probiotic beings lets us hope to solve, our alternate caring engagements no longer treat them as a resource and dissolves our self-given anthropocentric interests and sense of control and invite us to imagine a different world of caring and co-existence.
